# DUSP5 contributes to platinum resistance in ovarian cancer: single-cell discovery and functional validation

**DOI:** 10.3389/fphar.2026.1842271

**Published:** 2026-07-23

**Authors:** Chengfeng Liu, Wehua Li, Tingjun Liao, Zhenyi Li, Gang Huang, Qin Wang

**Affiliations:** 1 Department of Oncology, The Affiliated Traditional Chinese Medicine Hospital of Southwest Medical University, Luzhou, China; 2 Department of General Surgery, Luzhou People’s Hospital, Luzhou, China; 3 Sichuan Provincial Center for Gynecology and Breast Diseases (Gynecology), Affiliated Hospital of Southwest Medical University, Luzhou, China; 4 Clinical Medical College, Southwest Medical University, Luzhou, China

**Keywords:** DUSP5, multi-omics integration, ovarian cancer, platinum resistance, precision oncology, therapeutic target, tumor microenvironment

## Abstract

**Background:**

Platinum-based chemotherapy remains the cornerstone of ovarian cancer treatment, yet acquired resistance severely limits efficacy. Because platinum agents can also influence immunogenic cell death and tumor microenvironment (TME) remodeling, clarifying cellular pharmacological mechanisms of sensitivity and resistance within the ovarian cancer tumor ecosystem is important for understanding therapeutic failure.

**Methods:**

We integrated single-cell RNA-seq from treatment-naïve and post-neoadjuvant chemotherapy ovarian tumors with bulk multi-omics cohorts to map epithelial tumor heterogeneity, transcriptional reprogramming, pathway activation, immune infiltration, and inferred cell–cell communication networks. DUSP5 was identified as a candidate regulator linked to stress-adaptive programs. Functional validation included qPCR, proliferation, migration, colony-formation, carboplatin dose-response, and xenograft assays following stable DUSP5 knockdown.

**Results:**

Single-cell analysis revealed chemotherapy-associated epithelial states with enhanced stress, EMT, hypoxia, and inflammatory signatures. Elevated DUSP5 expression correlated with MAPK/JAK-STAT/TGF-β signaling, myeloid/stromal infiltration, and clinical outcome differences in independent cohorts. DUSP5 depletion suppressed proliferation and migration, amplified carboplatin-induced MAPK transcriptional responses and pro-apoptotic signaling (BAX/PUMA upregulation, BCL2 downregulation), reduced IC50 values in both OVCAR8 and SKOV3 cells, and significantly inhibited xenograft tumor growth.

**Conclusion:**

DUSP5 may contribute to platinum response and resistance by linking tumor-intrinsic adaptive programs with TME-associated features in ovarian cancer. These findings support DUSP5 as a candidate biomarker and therapeutic target that warrants further mechanistic and clinical validation.

## 1 Introduction

Ovarian cancer remains one of the most lethal gynecological malignancies worldwide and is characterized by high recurrence rates and poor long-term survival (Stewart et al., 2019). Despite advances in surgical management and systemic therapy, platinum-based chemotherapy continues to represent the cornerstone of first-line treatment for most patients with advanced disease (Webb and Jordan, 2017; Peng et al., 2022). Although many patients initially respond to platinum-based regimens, a considerable proportion ultimately develop treatment resistance, leading to tumor recurrence and disease progression (Eisenhauer, 2017). Platinum resistance therefore represents a major obstacle in the clinical management of ovarian cancer and remains a critical determinant of patient prognosis (Havasi et al., 2023). Understanding the molecular mechanisms that drive chemotherapy resistance is essential for improving therapeutic strategies and identifying new targets for intervention (Zhang et al., 2022).

Previous studies have shown that chemotherapy resistance arises through a combination of tumor-intrinsic adaptations and interactions with the surrounding tumor microenvironment. Tumor cells exposed to therapeutic stress can undergo profound transcriptional and phenotypic reprogramming, enabling them to survive cytotoxic pressure. Several biological processes have been implicated in this adaptive response, including activation of stress signaling pathways, epithelial–mesenchymal transition, metabolic reprogramming, and inflammatory signaling (Zhang et al., 2025; Leung et al., 2022). At the same time, increasing evidence suggests that chemotherapy can also reshape the tumor microenvironment. Changes in immune and stromal cell populations, extracellular-vesicle-mediated communication, broader immune context, and stromal remodeling may collectively contribute to tumor persistence and therapy resistance (Shen et al., 2022; Launonen et al., 2024; Gong et al., 2023; Yan et al., 2025; Chi et al., 2025; Li et al., 2023). These findings indicate that treatment resistance should be considered not only as a tumor cell–intrinsic phenomenon but also as a dynamic ecosystem process involving interactions between tumor cells and their microenvironment.

Recent advances in single-cell RNA sequencing have provided new opportunities to investigate tumor heterogeneity and treatment-associated cellular adaptation at unprecedented resolution (Yan et al., 2025; Izar et al., 2020; He et al., 2024). Compared with bulk transcriptomic analyses, single-cell approaches enable the identification of distinct tumor cell states and the reconstruction of transcriptional trajectories during tumor evolution and therapeutic response. Integrative strategies that combine single-cell transcriptomics with bulk multi-omics datasets and pathology-informed computational analyses further facilitate the discovery of molecular regulators associated with tumor plasticity, microenvironment remodeling, and therapy resistance (Izar et al., 2020; Wang et al., 2022). In ovarian cancer, emerging studies have begun to reveal complex transcriptional states within epithelial tumor cells, some of which appear to be linked to stress responses and inflammatory signaling following chemotherapy exposure (Kang et al., 2024). However, the key regulators that connect epithelial tumor cell plasticity with treatment-associated transcriptional programs and immune microenvironment changes remain incompletely understood.

In the present study, we performed integrative analyses combining single-cell transcriptomics and bulk multi-omics datasets to investigate chemotherapy-associated tumor adaptation in ovarian cancer. By analyzing single-cell RNA sequencing data from treatment-naive and post-neoadjuvant chemotherapy tumors, we characterized epithelial tumor cell heterogeneity and identified transcriptional states associated with chemotherapy exposure. Through integrated computational analyses, DUSP5 was identified as a candidate regulator linked to stress-associated epithelial programs (Zhao et al., 2023b; Zandi et al., 2022). Subsequent bulk transcriptomic analyses were used to evaluate the clinical relevance of DUSP5 and its associations with signaling pathways and tumor microenvironment features. Finally, functional experiments were performed to investigate the biological role of DUSP5 in ovarian cancer cell behavior and platinum sensitivity. Together, these analyses provide new insights into the transcriptional programs associated with chemotherapy adaptation and highlight DUSP5 as a potential regulator of platinum resistance in ovarian cancer.

## 2 Methods

### 2.1 Single-cell RNA sequencing data processing, integration, and cell type annotation

Single-cell RNA sequencing data were obtained from the Gene Expression Omnibus (GEO) under accession number GSE165897, which includes tumor samples from patients with high-grade serous ovarian cancer (HGSOC) collected before neoadjuvant chemotherapy (treatment-naive) and after neoadjuvant chemotherapy (post-NACT) (Zhang et al., 2022). Data preprocessing was performed using the Seurat package in R (Stuart et al., 2019). Cells were filtered based on quality control metrics including the number of detected genes (nFeature_RNA), total UMI counts (nCount_RNA), mitochondrial gene expression proportion (percent.mt), and hemoglobin gene expression proportion (percent.HB). Cells with low gene complexity or high mitochondrial gene content were excluded from downstream analysis. Following quality control, data were normalized using the LogNormalize method. Highly variable genes were identified and used for downstream analysis. After scaling, principal component analysis (PCA) was performed. To correct for batch effects across samples, Harmony was applied using sample identity as the integration variable (Korsunsky et al., 2019). UMAP dimensionality reduction and graph-based clustering were conducted based on the corrected low-dimensional embeddings. Major cell types were annotated based on canonical marker gene expression, enabling identification of epithelial tumor cells (EOC), immune cells, and stromal cells (Izar et al., 2020). The relative distribution of major cell populations across treatment stages was subsequently evaluated.

### 2.2 Identification and annotation of epithelial tumor subtypes

To characterize intratumoral heterogeneity, epithelial tumor cells were extracted from the integrated dataset and re-analyzed independently. Within the epithelial subset, highly variable genes were re-identified, followed by dimensionality reduction and graph-based clustering to resolve epithelial subpopulations. Based on differential gene expression profiles and functional enrichment patterns, epithelial clusters were consolidated into five biologically interpretable transcriptional states, namely, IFN_AntigenPresentation, Secretory_EpithelialLike, Stress_IEG_SenescenceLike, NormalLike_Differentiated, and Cycling_Proliferating. The spatial distribution of these epithelial states in low-dimensional embedding space was examined, and their proportional changes across treatment stages were systematically evaluated at both pooled-cell and patient levels.

### 2.3 Copy number variation inference

Copy number variation (CNV) inference was performed using the CopyKAT package based on raw count matrices (Gao et al., 2021). Cells were classified as aneuploid, diploid, or NA according to inferred CNV patterns. CNV results were used to support malignant attribution of epithelial subsets; however, diploid or NA epithelial cells were not automatically excluded from downstream epithelial-state analyses, because transcriptome-based CNV inference may incompletely capture malignant epithelial cells with weak or ambiguous CNV signals.

### 2.4 Quantification of epithelial functional states

To quantify functional heterogeneity among epithelial tumor cells, gene set–based module scores were calculated to represent multiple biological programs, including basal/progenitor characteristics, cell cycle activity, epithelial–mesenchymal transition (EMT), hypoxia response, and interferon-associated inflammatory response (Hänzelmann et al., 2013). Functional scores were subsequently compared across epithelial transcriptional states and between treatment stages to assess differences in program activation patterns.

### 2.5 Differential expression analysis using a pseudo-bulk strategy

To identify chemotherapy-associated transcriptional alterations, differential expression analysis was performed between treatment-naive and post-NACT epithelial cells using a pseudo-bulk approach (Squair et al., 2021). Gene expression counts were aggregated by patient and treatment stage to reduce statistical bias inherent to single-cell–level testing. Differential expression analysis was conducted using DESeq2 (Love et al., 2014). Chemotherapy-associated genes were considered significant using an FDR threshold of <0.05 and an absolute log2 fold-change threshold of >1, unless otherwise specified for exploratory visualization. Additionally, the directionality of gene expression changes was evaluated across patients to identify robust and consistent chemotherapy-associated genes.

### 2.6 Gene set enrichment analysis

Gene set enrichment analysis (GSEA) was performed using Hallmark gene sets (Liberzon et al., 2015; Subramanian et al., 2005). Genes were ranked based on statistical metrics derived from differential expression analysis. Enrichment scores were calculated to evaluate pathway-level activation or suppression across treatment stages within specific epithelial states.

### 2.7 Pseudotime trajectory analysis

To investigate dynamic transitions of epithelial tumor states, pseudotime trajectory analysis was conducted using Monocle2 (Qiu et al., 2017). Ordering genes were selected based on dispersion and biological relevance. The distribution of epithelial states and treatment stages along the pseudotime trajectory was analyzed. Chemotherapy-associated genes were mapped onto the trajectory to assess dynamic expression patterns during state transitions.

### 2.8 Cell–cell communication analysis

Intercellular communication networks were computationally inferred using the CellChat package (Jin et al., 2021). Cell type annotations were simplified to facilitate communication modeling. Balanced subsampling was performed to ensure comparability between treatment stages. Separate CellChat models were constructed for treatment-naive and post-NACT samples. Differences in inferred interaction number, interaction strength, and pathway-level information flow were compared across treatment stages, with particular attention to predicted communication between epithelial tumor states and immune or stromal cells.

### 2.9 Bulk transcriptomic validation and survival analysis of DUSP5

To validate the expression pattern and clinical relevance of DUSP5 identified from the single-cell analysis, multiple bulk transcriptomic datasets were analyzed (Goldman et al., 2020). RNA-seq data for ovarian cancer were obtained from TCGA-OV (n = 379) and normal ovarian tissues from the GTEx database (n = 88) through the UCSC Xena platform (tcga_RSEM_gene_tpm and gtex_RSEM_gene_tpm datasets). Expression values were transformed into Z-scores using (x − μ)/σ to minimize systematic differences between datasets, and samples with extreme values (Z-score < −3 or >3) were removed prior to analysis. Differential expression between tumor and normal tissues was assessed using the Wilcoxon rank-sum test, and the results were visualized using the ggplot2 package. The GSE26712 dataset (n = 185) was downloaded from the Gene Expression Omnibus (GEO) database and used for external validation of DUSP5 expression, and comparisons among ovarian cancer molecular subtypes were also visualized using ggplot2 (Cancer Genome Atlas Research Network, 2011). For prognostic analysis, three independent cohorts with available survival data were analyzed: GSE13876 (high DUSP5, n = 197; low DUSP5, n = 218), GSE30161 (high DUSP5, n = 17; low DUSP5, n = 37), and GSE49997 (high DUSP5, n = 93; low DUSP5, n = 101). Patients were stratified into high- and low-expression groups using cohort-specific optimal cutoffs determined by maximally selected rank statistics, and Kaplan–Meier survival curves were generated using the survival and survminer packages (Hothorn and Zeileis, 2008). Because optimal cutoffs may introduce selection bias, these analyses were interpreted as supportive clinical associations rather than definitive prognostic validation.

### 2.10 Pathway enrichment and functional state association analysis based on DUSP5 expression

To characterize the biological programs associated with DUSP5 in ovarian cancer, tumor samples from the bulk cohort were stratified into DUSP5-high and DUSP5-low groups by selecting the top 30% and bottom 30% of samples according to DUSP5 expression (z-score). Differential expression between the two groups was performed using the limma package, and genes were ranked by the direction and magnitude of differential expression (e.g., log2 fold change or moderated statistics) to generate an ordered list for enrichment analysis (Ritchie et al., 2015). Gene set enrichment analysis (GSEA) was then conducted with clusterProfiler using KEGG pathway gene sets, and enriched pathways were summarized by normalized enrichment score (NES) with significance assessed by permutation-based testing and multiple-testing correction; enrichment results were visualized as a two-sided bar/heatmap-style summary using ggplot2, with red indicating pathways enriched in the DUSP5-high group and blue indicating pathways enriched in the DUSP5-low group (Wu et al., 2021). In parallel, to quantify tumor functional phenotypes, 14 cancer functional state signatures curated from CancerSEA were scored using the GSVA package (z-score method), followed by z-normalization of signature scores across samples (Yuan et al., 2019); Pearson correlation was used to evaluate associations between DUSP5 expression and each functional state score, and scatterplots with fitted trends were generated using ggplot2.

### 2.11 Immune infiltration association analysis stratified by DUSP5 expression

To evaluate the relationship between DUSP5 and the immune microenvironment in ovarian cancer, immune infiltration estimates for TCGA-OV and multiple GEO ovarian cancer cohorts were retrieved from TIMER2.0, covering several deconvolution algorithms (e.g., TIMER, CIBERSORT/CIBERSORT-ABS, xCell, MCP-counter, quanTIseq, EPIC) (Li et al., 2020). For each cohort and algorithm, Spearman correlation was calculated between DUSP5 expression and inferred immune/stromal cell abundance, and significant associations (p < 0.05) were summarized as a correlation heatmap, with non-significant results marked accordingly. In addition, OV samples were divided into DUSP5-high and DUSP5-low groups using the median expression, and immune/stromal cell scores were compared between groups using the Wilcoxon rank-sum test; samples were displayed in order of increasing DUSP5 expression to visualize coordinated changes in the tumor microenvironment. Key cell-type correlations in OV were further summarized in a scatter/lollipop-style plot to highlight consistency across representative cell populations.

### 2.12 Cell lines and cell culture

The human ovarian surface epithelial cell line IOSE80 (RRID: CVCL_4884), human ovarian carcinoma cell lines OVCAR8 (RRID: CVCL_1629) and SKOV3 (RRID: CVCL_0532), as well as the human embryonic kidney cell line HEK293 (RRID: CVCL_0045), were obtained from the American Type Culture Collection (ATCC, Manassas, VA, USA). All cell lines were authenticated by short tandem repeat (STR) profiling and routinely tested negative for *mycoplasma* contamination prior to experimental use. IOSE80 cells were cultured in RPMI-1640 medium (Gibco, Cat. No. 11875093) supplemented with 10% fetal bovine serum (FBS; Gibco, Cat. No. 10099141C) and 1% penicillin–streptomycin solution (Gibco, Cat. No. 15140122). OVCAR8 and SKOV3 cells were maintained in DMEM (Gibco, Cat. No. 11965092) containing 10% FBS and antibiotics as described above. HEK293 cells were cultured in high-glucose DMEM (Gibco, Cat. No. 11965092) with 10% FBS. All cells were incubated at 37 °C in a humidified atmosphere with 5% CO_2_ and subcultured at approximately 70%–80% confluence using 0.25% trypsin–EDTA solution (Gibco, Cat. No. 25200072). Cells within 20 passages were used for all experiments.

### 2.13 Quantitative real-time PCR

Total RNA was isolated from cells with TRIzol™ reagent (Invitrogen, Thermo Fisher Scientific, Cat. No. 15596026). RNA yield and quality were assessed spectrophotometrically by determining A260/A280 ratios using a NanoDrop One instrument (Thermo Scientific). Complementary DNA was synthesized from 1 μg RNA using the PrimeScript™ RT reagent kit with gDNA Eraser (Takara, Cat. No. RR047A) to remove residual genomic DNA. Reverse transcription was conducted at 37 °C for 15 min followed by enzyme inactivation at 85 °C for 5 s. Quantitative amplification was performed with TB Green® Premix Ex Taq™ II (Takara, Cat. No. RR820A) on a QuantStudio™ 5 detection system (Applied Biosystems). Reactions were set up in a total volume of 20 μL, including TB Green master mix, gene-specific primers (0.2 μM final concentration each), and diluted cDNA template. Thermal cycling consisted of an initial denaturation step at 95 °C, followed by 40 amplification cycles under standard conditions. Product specificity was confirmed by melting curve profiling. Transcript abundance was normalized to GAPDH expression and calculated using the comparative Ct (ΔΔCt) approach. All measurements were conducted in technical triplicates with at least three independent biological repeats. The primer sequences used in this study were as follows:

GAPDH-F: GAC​AGT​CAG​CCG​CAT​CTT​CT.

GAPDH-R: GCG​CCC​AAT​ACG​ACC​AAA​TC.

FOS-F: CAG​ACT​ACG​AGG​CGT​CAT​CC.

FOS-R: AGT​TGG​TCT​GTC​TCC​GCT​TG.

EGR1-F: CAC​CTG​ACC​GCA​GAG​TCT​TT.

EGR1-R: CTG​ACC​AAG​CTG​AAG​AGG​GG.

JUN-F: CAG​CCA​GGT​CGG​CAG​TAT​AG.

JUN-R: GGA​CTC​TGC​CAC​TTG​TCT​CC.

BAX-F: TTC​ATC​CAG​GAT​CGA​GCA​GG.

BAX-R: TGT​CCA​GCC​CAT​GAT​GGT​TC.

PUMA-F: AGG​ATG​AAA​TTT​GGC​ATG​GGG.

PUMA-R: GCT​GCT​CCT​CTT​GTC​TCA​CA.

BCL2-F: AAA​AAT​ACA​ACA​TCA​CAG​AGG​AAG​T.

BCL2-R: TCC​CGG​TTA​TCG​TAC​CCT​GT.

DUSP5-F: GTG​CCT​ACT​GCA​CAT​TCC​CT.

DUSP5-R: TCC​CGA​GAA​CCT​ACC​CTG​AG.

### 2.14 Stable DUSP5 knockdown and validation

Short hairpin RNA (shRNA) sequences targeting human DUSP5 and a non-targeting control sequence were cloned into the pLKO.1-puro lentiviral vector (Addgene, Cat. No. 8453). Lentiviral particles were produced in HEK293 cells by co-transfecting the shRNA plasmid together with the packaging plasmids psPAX2 (Addgene, Cat. No. 12260) and pMD2.G (Addgene, Cat. No. 12259) using Lipofectamine™ 3000 (Invitrogen, Thermo Fisher Scientific, Cat. No. L3000015). Viral supernatants were collected 48–72 h after transfection, filtered through a 0.45 μm membrane, and used immediately or stored at −80 °C. OVCAR8 and SKOV3 cells were exposed to viral supernatants in the presence of 8 μg/mL polybrene (Sigma-Aldrich, Cat. No. TR-1003-G) to enhance transduction efficiency. After 24 h, the medium was replaced with fresh complete medium. Puromycin (Sigma-Aldrich, Cat. No. P8833) was applied for antibiotic selection at concentrations of 1–2 μg/mL for OVCAR8 and 2–3 μg/mL for SKOV3, based on prior kill curve determination. Stable pools were maintained under continuous puromycin selection. Knockdown efficiency was assessed at mRNA levels. DUSP5 transcript levels were measured by quantitative real-time PCR as described above. Stable clones exhibiting consistent suppression of DUSP5 expression were selected for subsequent experiments.

### 2.15 Colony formation assay

For assessment of long-term proliferative capacity, cells were seeded into six-well plates (Corning, Cat. No. 3516) at a density of 600 cells per well and allowed to adhere overnight. The following day, cells were divided into treatment and control groups. The treatment group received 10 μM carboplatin (Selleck Chemicals, Cat. No. S1215) in complete medium for 24 h, whereas control cells were maintained in parallel under identical conditions without drug exposure. After treatment, the medium was replaced with fresh drug-free complete medium for all groups. Cells were cultured for 12–14 days with medium refreshed every 3 days until visible colonies developed. At the endpoint, colonies were fixed in 4% paraformaldehyde (Beyotime, Cat. No. P0099) for 20 min and stained with 0.1% crystal violet (Solarbio, Cat. No. G1062). Colonies containing more than 50 cells were considered viable colonies and were counted manually or quantified using ImageJ software (NIH). Each experimental condition was performed in triplicate wells, and the assay was independently repeated at least three times.

### 2.16 CCK-8 assay

Cell viability was evaluated using the Cell Counting Kit-8 (CCK-8) assay (Dojindo, Cat. No. CK04). Cells were dispensed into 96-well plates (Corning, Cat. No. 3599) at 2,000–3,000 cells per well in 100 μL complete medium and allowed to attach overnight. For proliferation curves, absorbance was recorded at the indicated time points (0, 24, 48, 72, and 96 h). For drug-response experiments, cells were exposed to carboplatin (Selleck Chemicals, Cat. No. S1215) at serial concentrations (0–80 μM, as indicated in the figure) for 72 h. At each readout, 10 μL of CCK-8 reagent was added directly to each well, followed by incubation for 1–2 h at 37 °C protected from light. Optical density was measured at 450 nm using a microplate reader (BioTek Synergy H1, Agilent). Background signals were corrected using medium-only wells. Cell viability was expressed as a percentage relative to the untreated control. Dose–response curves and IC50 values were determined by nonlinear regression (log[inhibitor] versus normalized response) in GraphPad Prism (version 9.0). Each condition was tested in 3 technical replicates, and experiments were independently repeated at least three times.

### 2.17 Wound-healing migration assay

Cell migratory capacity was assessed using a wound-healing assay. Briefly, shNC and shDUSP5 stable cells were seeded into 6-well plates (Corning, Cat. No. 3516) and grown to near confluence. A straight scratch was generated across the monolayer using a sterile 200 μL pipette tip. Detached cells were removed by gentle washing with phosphate-buffered saline (PBS; Gibco, Cat. No. 10010023). To minimize the contribution of proliferation, cells were maintained in serum-reduced medium (DMEM containing 1% FBS) during the migration period. Images were captured immediately after scratching (0 h) and again at 48 h using an inverted microscope at the same marked positions. Wound closure was quantified by measuring the scratch area (or wound width) at each time point using ImageJ software (NIH). Migration rate was calculated as: (A0 − At)/A0 × 100%, where A0 and At represent the wound area at 0 h and 48 h, respectively. Each condition was performed in triplicate, and experiments were independently repeated at least three times.

### 2.18 Xenograft tumor model

All animal experiments were conducted in accordance with institutional guidelines and approved by the Institutional Animal Care and Use Committee. Female BALB/c nude mice (4–6 weeks old) were purchased from an accredited animal facility and housed under specific pathogen-free conditions. Stable shNC and shDUSP5 OVCAR8 cells in logarithmic growth phase were harvested, washed twice with PBS, and resuspended in serum-free medium. A total of 5 × 10^6 cells in 100 μL were subcutaneously injected into the right flank of each mouse, with six mice included in each group. Tumor growth was monitored every 3 days using a digital caliper according to a predefined measurement schedule. Tumor volume was calculated using the formula: volume = (length × width^2^)/2. Mice were observed for general health and body weight throughout the experiment. At the experimental endpoint, mice were humanely euthanized by 40% CO_2_ inhalation. Tumors were excised, photographed, and weighed. Tumor growth curves and final tumor weights were used to evaluate the effect of DUSP5 knockdown on ovarian cancer progression *in vivo*.

### 2.19 Statistical analysis

Statistical analyses were performed using R software (version 4.2.0) and GraphPad Prism (version 9.0). For bioinformatic analyses, differential expression was conducted using DESeq2 or limma as described above, and correlation analyses were performed using Pearson or Spearman methods. Survival differences were evaluated using Kaplan-Meier analysis with the log-rank test. For *in vitro* assays, results are presented as mean ± SD from at least three independent experiments; xenograft data are presented as mean ± SD based on six mice per group. Comparisons between two groups were performed using a two-tailed unpaired Student’s t-test, while comparisons among multiple groups were analyzed using one-way ANOVA followed by Tukey’s *post hoc* test. Tumor growth curves were analyzed using two-way ANOVA. Dose–response curves and IC50 values were calculated by nonlinear regression analysis. A two-sided P value <0.05 was considered statistically significant.

## 3 Results

### 3.1 Global single-cell landscape of ovarian cancer before and after neoadjuvant chemotherapy

**FIGURE 1:**
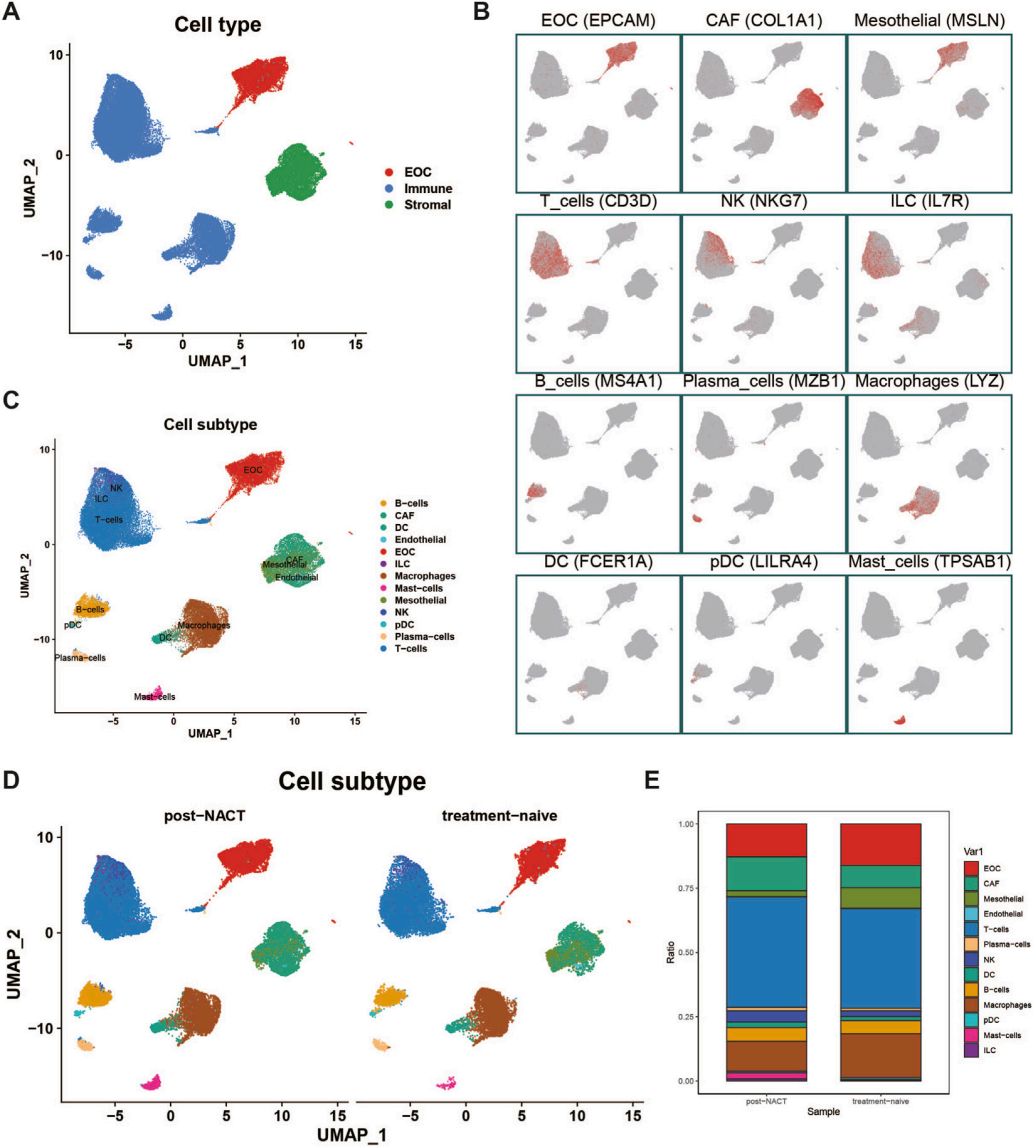
Single-cell landscape of ovarian cancer tumors before and after neoadjuvant chemotherapy. **(A)** UMAP visualization of integrated single-cell data showing major cell types. **(B)** Expression of representative marker genes supporting cell type annotation. **(C)** Distribution of annotated cell subtypes in UMAP space. **(D)** UMAP stratified by treatment stage (treatment-naive and post-NACT). **(E)** Relative proportions of annotated cell subtypes across treatment stages.

Integrated single-cell analysis identified epithelial tumor cells, immune cells, and stromal cells as the principal components of the ovarian tumor microenvironment (Figure 1A). Canonical marker gene expression supported accurate cell type annotation (Figure 1B). Distinct cell populations were well separated in UMAP space, reflecting the cellular heterogeneity of the tumor microenvironment (Figure 1C). Comparison between treatment-naive and post-NACT samples revealed that overall cellular architecture remained largely preserved (Figure 1D). However, relative proportions of specific cell types exhibited moderate changes across treatment stages (Figure 1E), suggesting selective remodeling of certain cellular compartments following chemotherapy.

### 3.2 Transcriptional heterogeneity of epithelial tumor cells and identification of major tumor states

**FIGURE 2:**
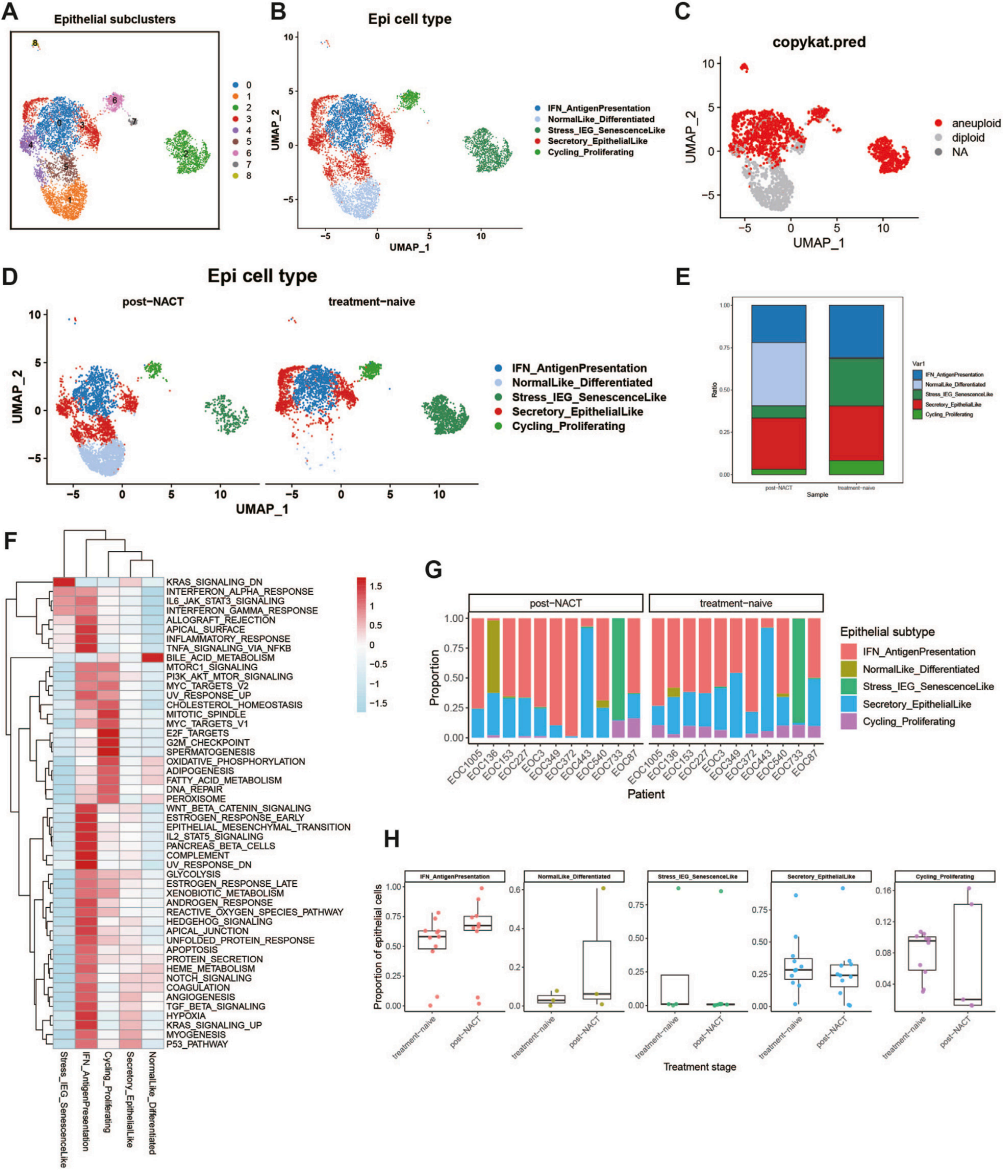
Transcriptional heterogeneity and epithelial tumor states. **(A)** Re-clustering of epithelial tumor cells in UMAP space. **(B)** Consolidation of epithelial subclusters into five transcriptional states. **(C)** UMAP visualization of CopyKAT-inferred CNV status, including aneuploid, diploid, and NA classifications. **(D)** UMAP visualization of epithelial cells stratified by treatment stage. **(E)** Proportional changes of epithelial states across treatment stages. **(F)** Hallmark pathway enrichment across epithelial states. **(G,H)** Patient-level distribution and comparative analysis of epithelial state proportions.

Re-clustering of epithelial tumor cells revealed multiple transcriptionally distinct subclusters (Figure 2A). Based on differential expression patterns and functional annotation, these subclusters were consolidated into five epithelial transcriptional states (Figure 2B). IFN_AntigenPresentation cells were enriched for interferon-response and antigen-presentation programs, Secretory_EpithelialLike cells showed epithelial/secretory features and chemotherapy-associated transcriptional changes, Stress_IEG_SenescenceLike cells displayed immediate-early gene and stress-response signatures, NormalLike_Differentiated cells exhibited more differentiated epithelial features, and Cycling_Proliferating cells were enriched for cell-cycle programs. Copy number variation inference demonstrated differential distributions of aneuploid, diploid, and NA cells among epithelial subsets (Figure 2C), supporting epithelial heterogeneity while avoiding overinterpretation of CNV status alone. UMAP visualization stratified by treatment stage indicated redistribution of epithelial states following NACT (Figure 2D). Proportional analysis revealed dynamic shifts in epithelial state composition across treatment stages (Figure 2E). Hallmark pathway enrichment further confirmed distinct pathway activation patterns among epithelial states (Figure 2F). Inter-patient variability was also evident at the individual level, as shown by patient-wise epithelial state proportions and comparative analyses (Figures 2G,H).

### 3.3 Remodeling of intercellular communication networks following chemotherapy

**FIGURE 3:**
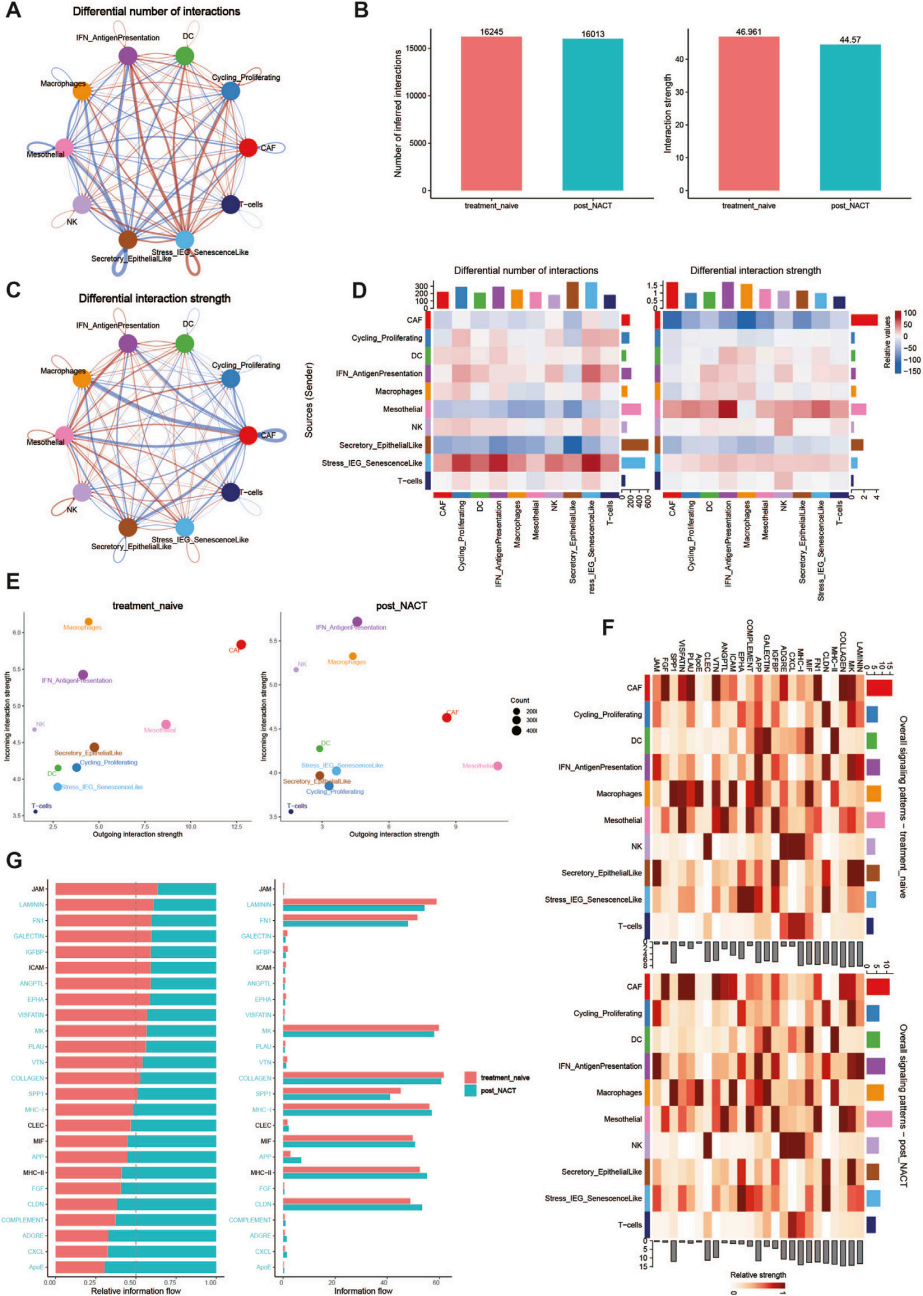
Chemotherapy-associated remodeling of inferred cell–cell communication networks. **(A)** Differential interaction network between treatment stages. **(B)** Comparison of overall inferred interaction number and strength. **(C)** Differential interaction-strength network between treatment stages. **(D)** Heatmaps of differential interaction number and interaction strength across cell-type pairs. **(E)** Outgoing and incoming signaling activity by cell type. **(F)** Overall signaling-pattern heatmaps for treatment-naive and post-NACT samples. **(G)** Relative and absolute pathway-level information flow comparison between treatment stages.

CellChat analysis inferred treatment-associated changes in intercellular communication networks. Network visualization demonstrated alterations in predicted interaction patterns between cell types across treatment stages (Figure 3A). While overall inferred interaction number and interaction strength remained comparable between treatment-naive and post-NACT conditions (Figure 3B), specific source–target relationships exhibited differential interaction intensity (Figures 3C,D). Analysis of outgoing and incoming signaling further highlighted cell-type–specific changes in communication roles (Figure 3E). Overall signaling-pattern heatmaps and pathway-level information-flow analyses identified multiple signaling programs with differential activity between treatment stages (Figures 3F,G), suggesting chemotherapy-associated remodeling of the tumor microenvironment communication landscape. These results should be interpreted as computationally inferred communication patterns rather than direct evidence of functional ligand–receptor signaling.

### 3.4 Chemotherapy-associated transcriptional reprogramming within epithelial subtypes

**FIGURE 4:**
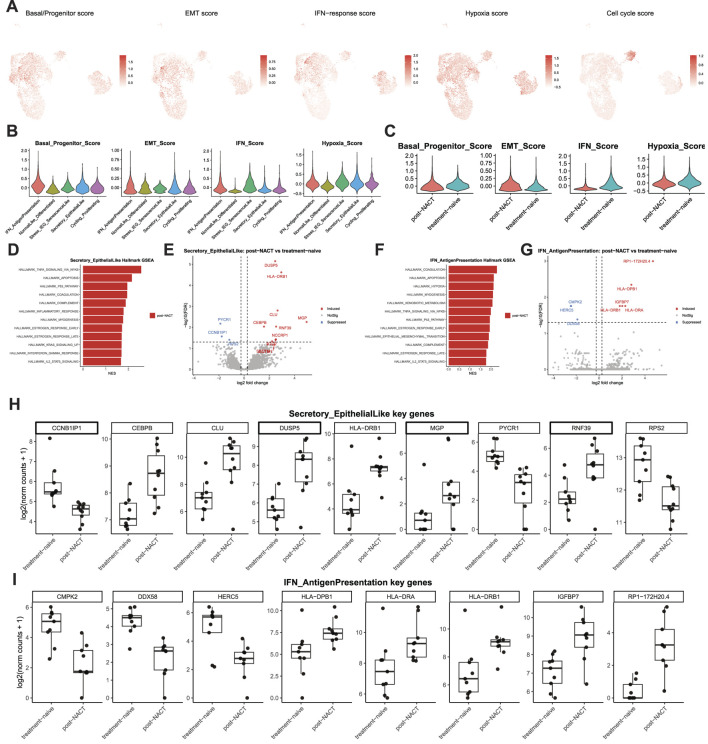
Functional programs and chemotherapy-associated transcriptional alterations. **(A)** Distribution of functional module scores in UMAP space. **(B)** Comparison of functional scores across epithelial states. **(C)** Treatment-stage comparison of functional programs. **(D–G)** GSEA and volcano plots of differential expression in Secretory_EpithelialLike and IFN_AntigenPresentation subsets. **(H,I)** Patient-level expression of key chemotherapy-associated genes.

Functional scoring demonstrated that epithelial states were characterized by distinct biological programs (Figures 4A,B). Comparison across treatment stages revealed alterations in selected functional axes following chemotherapy (Figure 4C). Within Secretory_EpithelialLike and IFN_AntigenPresentation subsets, pseudo-bulk differential expression analysis identified chemotherapy-associated genes between post-NACT and treatment-naive conditions, visualized by volcano plots (Figures 4E,G). Consistently, GSEA revealed pathway-level transcriptional reprogramming in these epithelial states (Figures 4D,F). Key differentially expressed genes exhibited consistent expression trends at the patient level (Figures 4H,I), supporting robust chemotherapy-associated transcriptional alterations.

### 3.5 Pseudotime trajectory analysis reveals dynamic redistribution of epithelial states

**FIGURE 5:**
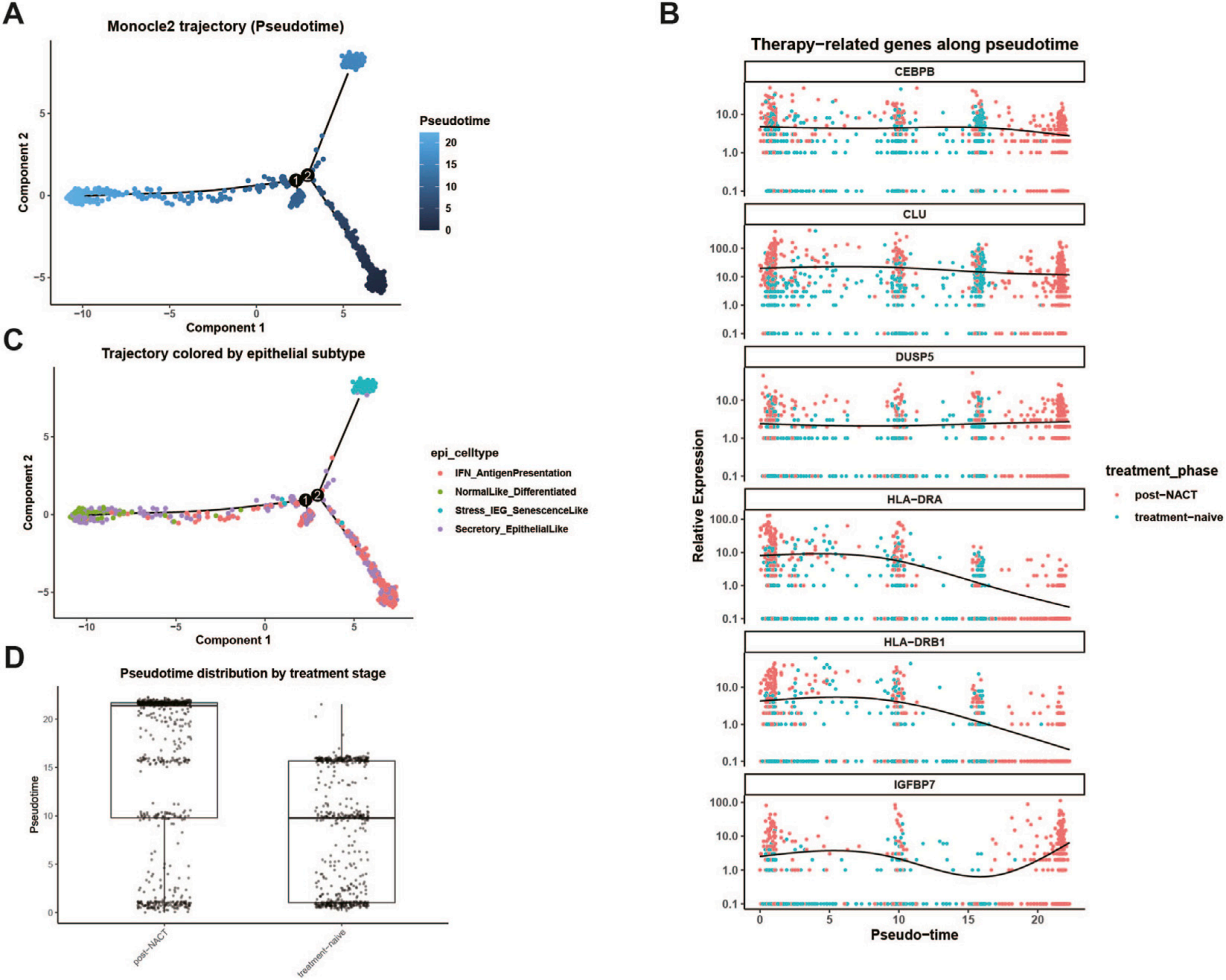
Pseudotime trajectory analysis of epithelial tumor states. **(A)** Monocle2 pseudotime trajectory of epithelial tumor cells. **(B)** Dynamic expression of chemotherapy-associated genes along pseudotime. **(C)** Distribution of epithelial states along the trajectory. **(D)** Distribution of treatment stages along pseudotime.

Pseudotime trajectory analysis revealed a continuous transcriptional landscape among epithelial tumor cells (Figure 5A). Distinct epithelial states occupied different regions along the trajectory (Figure 5C), indicating a transcriptional continuum rather than discrete compartments. Comparison of treatment stages along pseudotime demonstrated redistribution of cells following chemotherapy (Figure 5D). Chemotherapy-associated genes displayed dynamic expression patterns across the trajectory (Figure 5B), consistent with transcriptional reprogramming accompanying epithelial state transitions.

### 3.6 Bulk transcriptomic validation and prognostic relevance of DUSP5 in ovarian cancer

**FIGURE 6:**
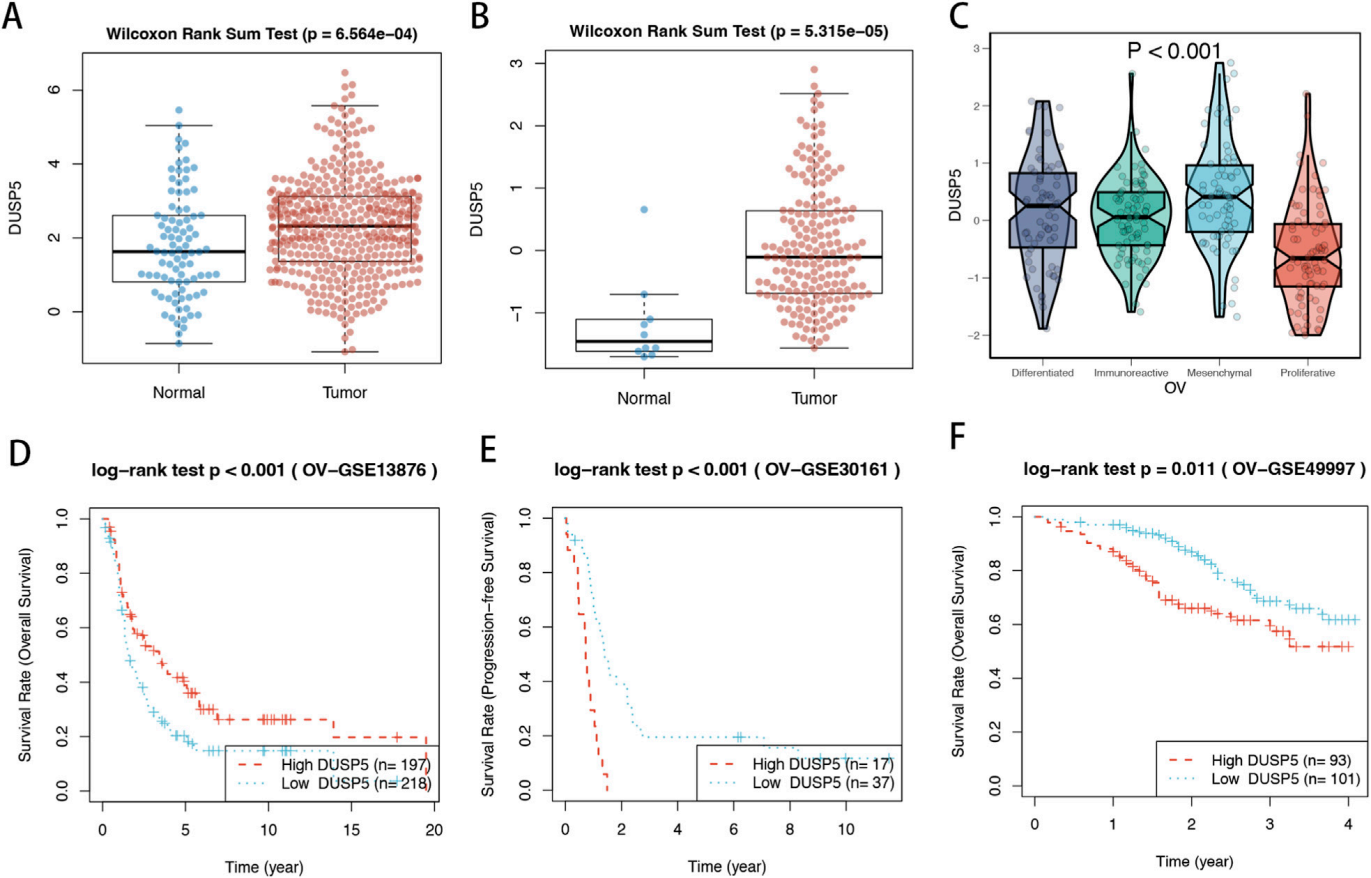
Expression pattern and clinical outcome associations of DUSP5 in ovarian cancer. **(A)** Differential expression of DUSP5 between ovarian tumor tissues and normal ovarian tissues based on integrated TCGA and GTEx datasets. **(B)** Validation of DUSP5 expression in ovarian cancer tissues using the GSE26712 dataset. **(C)** Distribution of DUSP5 expression across ovarian cancer molecular subtypes. **(D)** Kaplan–Meier overall survival analysis of DUSP5 expression in the GSE13876 cohort. **(E)** Kaplan–Meier progression-free survival analysis of DUSP5 expression in the GSE30161 cohort. **(F)** Kaplan–Meier overall survival analysis of DUSP5 expression in the GSE49997 cohort.

Based on the single-cell analyses described above, DUSP5 was selected for further investigation because several lines of evidence converged on this gene. DUSP5 was identified among chemotherapy-associated genes in epithelial tumor states, particularly within the Secretory_EpithelialLike compartment, and showed patient-level treatment-associated expression changes. In addition, as a member of the MAPK phosphatase family involved in ERK signaling regulation, DUSP5 has been implicated in stress-induced transcriptional responses and signaling pathway remodeling, suggesting potential relevance to chemotherapy-associated tumor adaptation. To further evaluate its expression pattern, bulk transcriptomic datasets were analyzed. Analysis of integrated TCGA and GTEx datasets showed differential expression of DUSP5 between ovarian tumor tissues and normal ovarian tissues (Figure 6A). Consistent expression patterns were observed in the independent GSE26712 dataset (Figure 6B). Further analysis revealed that DUSP5 expression varied across ovarian cancer molecular subtypes (Figure 6C). To assess the clinical relevance of DUSP5, survival analyses were performed in multiple independent cohorts. Kaplan-Meier analysis demonstrated an association between DUSP5 expression and overall survival in the GSE13876 cohort (Figure 6D). In the GSE30161 cohort, DUSP5 expression was associated with progression-free survival (Figure 6E). A similar association with overall survival was observed in the GSE49997 dataset (Figure 6F). Given the use of cohort-specific optimal cutoffs, these survival findings were considered supportive clinical associations.

### 3.7 DUSP5-associated pathway remodeling and functional state signatures in ovarian cancer

**FIGURE 7:**
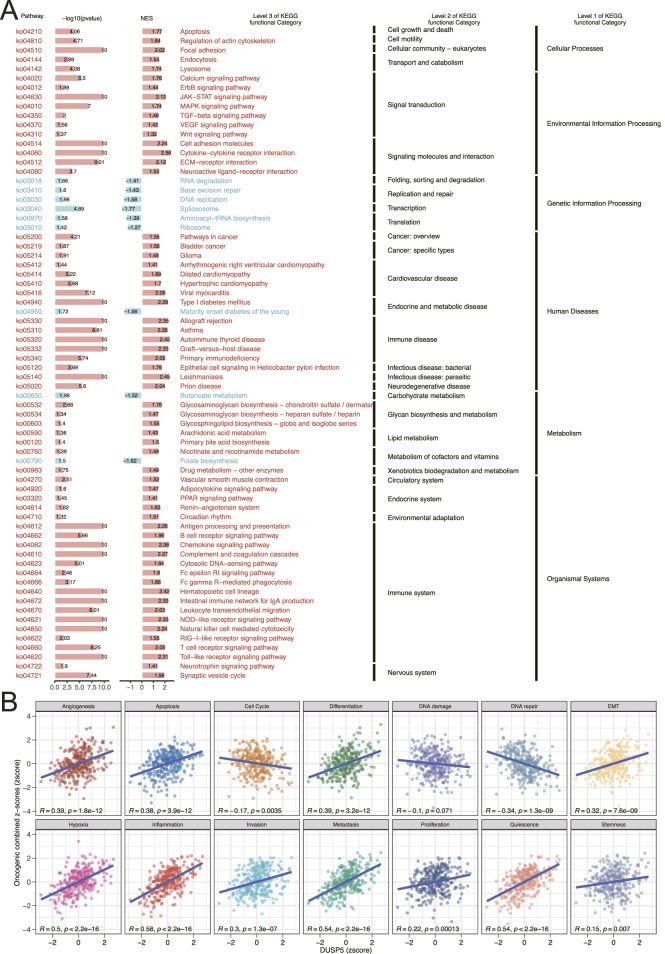
Pathway enrichment and functional state associations stratified by DUSP5 expression in ovarian cancer. **(A)** GSEA results comparing DUSP5-high versus DUSP5-low tumors. Red indicates pathways significantly enriched in the DUSP5-high group, while blue indicates pathways enriched in the DUSP5-low group; bars summarize pathway significance (−log10 adjusted p value) and normalized enrichment score (NES), with KEGG hierarchical categories shown on the right. **(B)** Associations between DUSP5 expression (z-score) and 14 CancerSEA functional state scores. Each panel shows a scatterplot of DUSP5 expression versus the corresponding functional state score with Pearson correlation coefficient (R) and p value.

To further explore the biological programs associated with DUSP5 expression in ovarian cancer, pathway enrichment and functional state analyses were performed by stratifying tumors into DUSP5-high and DUSP5-low groups. Gene set enrichment analysis revealed that tumors with high DUSP5 expression exhibited coordinated enrichment of pathways related to cell–matrix interaction and signal transduction, including focal adhesion, regulation of actin cytoskeleton, MAPK signaling, JAK–STAT signaling, TGF-β signaling, and VEGF signaling, together with immune-related pathways such as antigen processing and presentation (Figure 7A). In contrast, tumors with low DUSP5 expression were preferentially enriched for pathways involved in core cellular biosynthetic and proliferative processes, including ribosome, spliceosome, proteasome, DNA replication, and aminoacyl-tRNA biosynthesis (Figure 7A). Consistent with these pathway-level differences, functional state analysis demonstrated that DUSP5 expression was associated with distinct tumor phenotypic programs. DUSP5 expression showed positive correlations with functional states related to hypoxia, inflammation, epithelial–mesenchymal transition (EMT), metastasis, and cellular quiescence, whereas cell cycle activity and DNA repair-associated states showed negative correlations (Figure 7B). Together, these results indicate that DUSP5 expression in ovarian cancer is linked to stress-adaptive, signaling-driven, and microenvironment-associated functional programs, alongside reduced cell-cycle and repair-associated features.

### 3.8 Association between DUSP5 expression and immune infiltration in ovarian cancer

**FIGURE 8:**
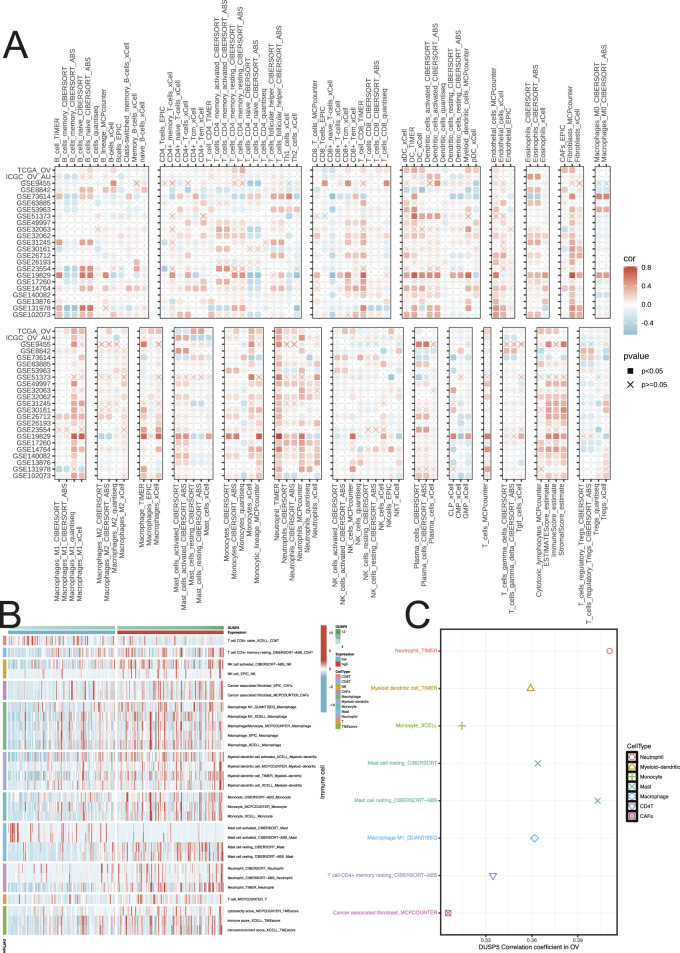
DUSP5-associated immune infiltration patterns in ovarian cancer. **(A)** Heatmap summarizing Spearman correlations between DUSP5 expression and inferred immune/stromal cell abundance across multiple OV cohorts and deconvolution algorithms; color indicates correlation direction and magnitude, and non-significant associations are marked. **(B)** Heatmap showing differences in estimated microenvironment components between DUSP5-low and DUSP5-high groups in OV, with samples ordered by increasing DUSP5 expression; color intensity reflects inferred cell abundance. **(C)** Summary plot of correlation coefficients between DUSP5 expression and representative immune/stromal cell types in OV, highlighting the relative strength of associations across key populations.

To explore the relationship between DUSP5 expression and the immune microenvironment, immune infiltration profiles were analyzed using multiple deconvolution algorithms. Correlation analysis showed that the association between DUSP5 expression and immune cell abundance varied across cell types and computational methods (Zhao et al., 2023a; Huang et al., 2023). Notably, myeloid-lineage immune cells exhibited the most consistent correlations with DUSP5 expression, including neutrophils, monocytes, and macrophage-related signatures. In contrast, lymphocyte populations generally displayed weaker or algorithm-dependent associations. These patterns suggest that DUSP5 expression may be preferentially linked to innate immune components of the tumor microenvironment rather than adaptive immune infiltration (Figure 8A). To further evaluate whether immune infiltration patterns differ according to DUSP5 expression levels, ovarian cancer samples were stratified into DUSP5-high and DUSP5-low groups. Comparison of immune cell abundance revealed significant differences in multiple immune and stromal components between the two groups. In particular, the DUSP5-high group showed increased infiltration of neutrophils, macrophage-associated cells, mast cells, and cancer-associated fibroblasts, whereas these components were relatively lower in the DUSP5-low group. These results indicate that tumors with elevated DUSP5 expression tend to exhibit a microenvironment enriched in inflammatory and stromal elements (Figure 8B). To summarize the overall strength of these associations, correlation coefficients between DUSP5 expression and representative immune cell populations were further visualized. Several immune and stromal cell types demonstrated moderate positive correlations with DUSP5 expression, with Spearman coefficients generally ranging from approximately 0.31–0.40. These correlations were particularly evident for neutrophils, monocytes, macrophage-related signatures, mast cells, and fibroblast-associated components, indicating that DUSP5 expression is linked to coordinated changes in multiple microenvironmental compartments rather than a single immune cell type (Figure 8C).

### 3.9 DUSP5 promotes proliferation and migration of ovarian cancer cells

**FIGURE 9:**
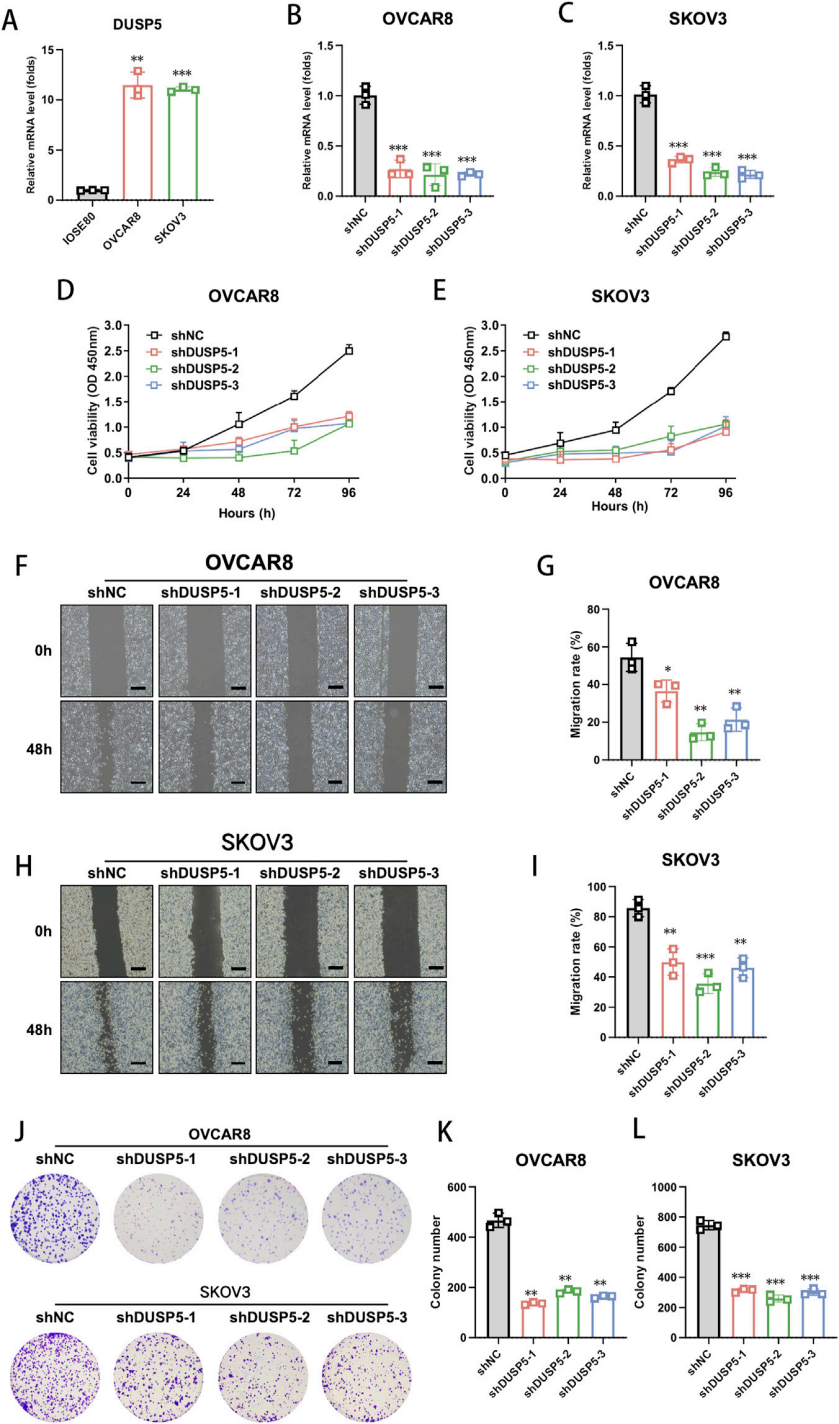
DUSP5 promotes proliferation and migration of ovarian cancer cells. **(A)** Relative mRNA expression of DUSP5 in normal ovarian epithelial cells (IOSE80) and ovarian cancer cell lines (OVCAR8 and SKOV3) determined by qPCR. **(B,C)** Validation of stable DUSP5 knockdown in ovarian cancer cells. Relative DUSP5 mRNA expression levels were measured in shNC and three independent shDUSP5 stable clones in OVCAR8 **(B)** and SKOV3 **(C)** cells. **(D,E)** Cell proliferation analysis using CCK-8 assays in shNC and shDUSP5 stable cells in OVCAR8 **(D)** and SKOV3 **(E)** cells. **(F–I)** Wound-healing migration assays in ovarian cancer cells. Representative images of wound closure at 0 h and 48 h in OVCAR8 **(F)** and SKOV3 **(H)** cells, with quantitative analysis of migration rates shown in **(G,I)**. **(J–L)** Colony formation assays evaluating clonogenic growth in ovarian cancer cells. Representative colony images **(J)** and quantification of colony numbers in OVCAR8 **(K)** and SKOV3 **(L)** cells. Data are presented as mean ± SD (n = 3). *P < 0.05, **P < 0.01, ***P < 0.001 versus shNC.

To investigate the biological role of DUSP5 in ovarian cancer, we first examined its expression in ovarian cancer cell lines. Quantitative real-time PCR analysis showed that DUSP5 mRNA levels were significantly higher in ovarian cancer cells (OVCAR8 and SKOV3) compared with normal ovarian epithelial cells (IOSE80) (Figure 9A). To further explore the functional significance of DUSP5, stable DUSP5 knockdown cell lines were generated using three independent shRNAs in both OVCAR8 and SKOV3 cells. qPCR validation confirmed that all three shDUSP5 constructs effectively reduced DUSP5 expression compared with the shNC control (Figures 9B,C). Functional assays demonstrated that DUSP5 depletion markedly suppressed ovarian cancer cell proliferation, as indicated by significantly reduced cell viability in CCK-8 assays in both OVCAR8 and SKOV3 cells (Figures 9D,E). We next assessed the impact of DUSP5 on cell migration using wound-healing assays. Representative images and quantitative analysis revealed that silencing DUSP5 significantly impaired the migratory ability of ovarian cancer cells, as shown by reduced wound closure rates in shDUSP5 cells compared with shNC controls (Figures 9F–I). In addition, colony formation assays demonstrated that DUSP5 knockdown substantially decreased the clonogenic capacity of ovarian cancer cells, with markedly fewer colonies observed in shDUSP5 groups in both OVCAR8 and SKOV3 cells (Figures 9J–L). Collectively, these results indicate that DUSP5 promotes proliferation, migration, and clonogenic growth of ovarian cancer cells, suggesting that DUSP5 may function as a tumor-promoting factor in ovarian cancer.

### 3.10 DUSP5 knockdown enhances carboplatin sensitivity in ovarian cancer cells

**FIGURE 10:**
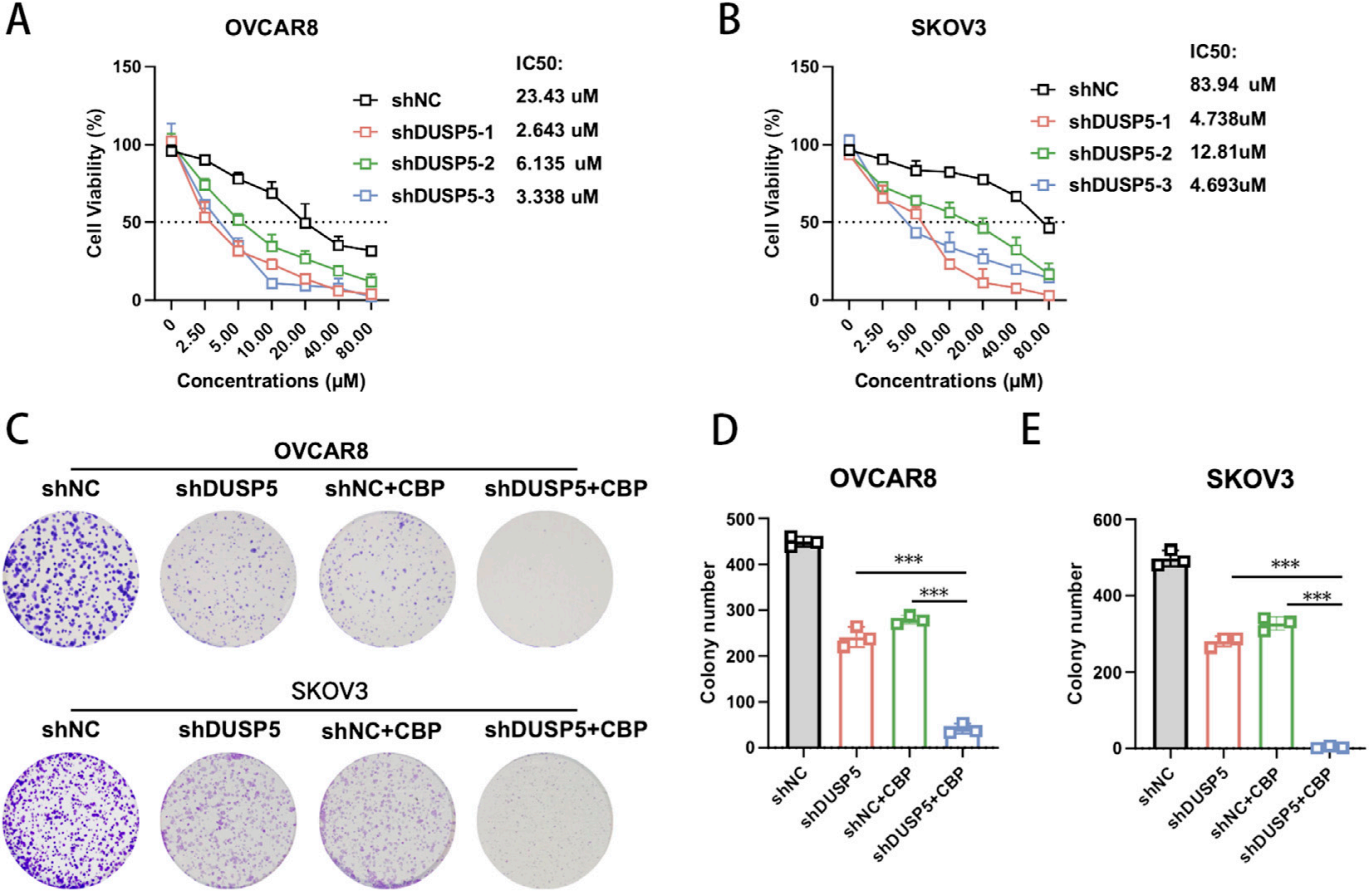
DUSP5 knockdown enhances carboplatin sensitivity in ovarian cancer cells. **(A,B)** Dose–response curves of carboplatin in shNC and shDUSP5 stable clones in OVCAR8 **(A)** and SKOV3 **(B)** cells determined by CCK-8 assays. DUSP5 knockdown significantly reduced IC50 values compared with shNC controls. **(C)** Representative colony formation images of shNC and shDUSP5 cells treated with or without carboplatin (CBP) in OVCAR8 and SKOV3 cells. **(D,E)** Quantification of colony numbers in OVCAR8 **(D)** and SKOV3 **(E)** cells under different treatment conditions. Combined DUSP5 knockdown and carboplatin treatment resulted in a significantly greater reduction in clonogenic survival compared with either treatment alone. Data are presented as mean ± SD (n = 3). ***P < 0.001.

To determine whether DUSP5 influences the response of ovarian cancer cells to platinum-based chemotherapy, we evaluated carboplatin sensitivity following DUSP5 knockdown. Dose–response curves generated from CCK-8 assays showed that silencing DUSP5 markedly increased the sensitivity of ovarian cancer cells to carboplatin, as evidenced by a substantial reduction in IC50 values in both OVCAR8 and SKOV3 cells compared with shNC controls (Figures 10A,B). In OVCAR8 cells, the IC50 decreased from 23.43 μM in shNC cells to 2.643, 6.135, and 3.338 μM in the three shDUSP5 groups. In SKOV3 cells, the IC50 decreased from 83.94 μM in shNC cells to 4.738, 12.81, and 4.693 μM in the three shDUSP5 groups. To further assess the impact of DUSP5 on clonogenic survival under chemotherapy stress, colony formation assays were performed in the presence or absence of carboplatin. Representative colony images and quantitative analyses demonstrated that both DUSP5 knockdown and carboplatin treatment individually reduced colony formation, while the combined treatment produced a markedly greater suppression of clonogenic growth in ovarian cancer cells (Figures 10C–E). These findings indicate that DUSP5 depletion potentiates the cytotoxic effects of carboplatin, suggesting that DUSP5 contributes to platinum resistance in ovarian cancer cells.

### 3.11 DUSP5 knockdown enhances MAPK transcriptional responses and promotes pro-apoptotic gene expression under carboplatin treatment

**FIGURE 11:**
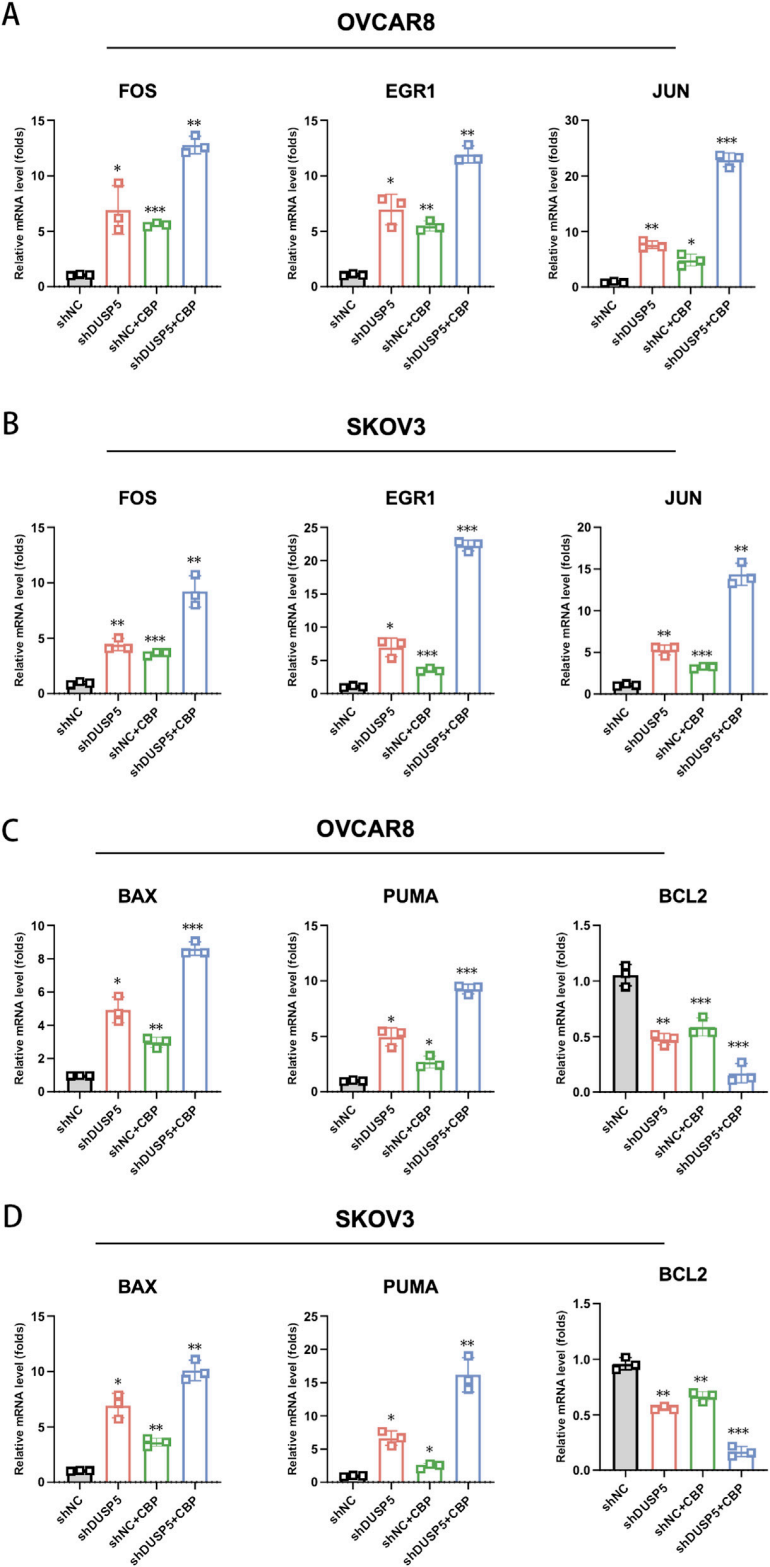
DUSP5 depletion enhances carboplatin-induced MAPK signaling and pro-apoptotic transcriptional responses in ovarian cancer cells. **(A,B)** Relative mRNA expression of MAPK/ERK downstream transcriptional targets FOS, EGR1, and JUN measured by qPCR in OVCAR8 **(A)** and SKOV3 **(B)** cells treated with or without carboplatin (CBP). DUSP5 knockdown enhanced carboplatin-induced transcriptional activation compared with shNC controls. **(C,D)** Relative mRNA expression of apoptosis-related genes BAX, PUMA, and BCL2 measured by qPCR in OVCAR8 **(C)** and SKOV3 **(D)** cells treated with or without carboplatin. DUSP5 depletion increased pro-apoptotic gene expression (BAX and PUMA) and reduced anti-apoptotic gene expression (BCL2) compared with control cells. Data are presented as mean ± SD (n = 3). *P < 0.05, **P < 0.01, ***P < 0.001.

To explore the potential molecular mechanism underlying the enhanced carboplatin sensitivity observed after DUSP5 depletion, we examined the transcriptional responses of MAPK/ERK downstream target genes. qPCR analysis revealed that carboplatin treatment significantly induced the expression of canonical MAPK transcriptional targets, including FOS, EGR1, and JUN, in ovarian cancer cells. Notably, DUSP5 knockdown further amplified these carboplatin-induced transcriptional responses in both OVCAR8 and SKOV3 cells (Figures 11A,B). Given that platinum-based chemotherapy primarily induces DNA damage–mediated apoptosis, we next assessed the transcriptional changes of apoptosis-related genes. qPCR results showed that carboplatin treatment increased the expression of pro-apoptotic genes BAX and PUMA while reducing the anti-apoptotic gene BCL2, and these effects were further enhanced by DUSP5 depletion in both ovarian cancer cell lines (Figures 11C,D). Specifically, DUSP5 knockdown markedly increased the expression of BAX and PUMA, while BCL2 expression was significantly reduced, indicating enhanced apoptotic signaling. Together, these findings suggest that DUSP5 depletion amplifies carboplatin-induced MAPK transcriptional activation and promotes pro-apoptotic transcriptional programs, thereby contributing to increased platinum sensitivity in ovarian cancer cells.

### 3.12 DUSP5 knockdown suppresses ovarian cancer tumor growth *in vivo*

**FIGURE 12:**
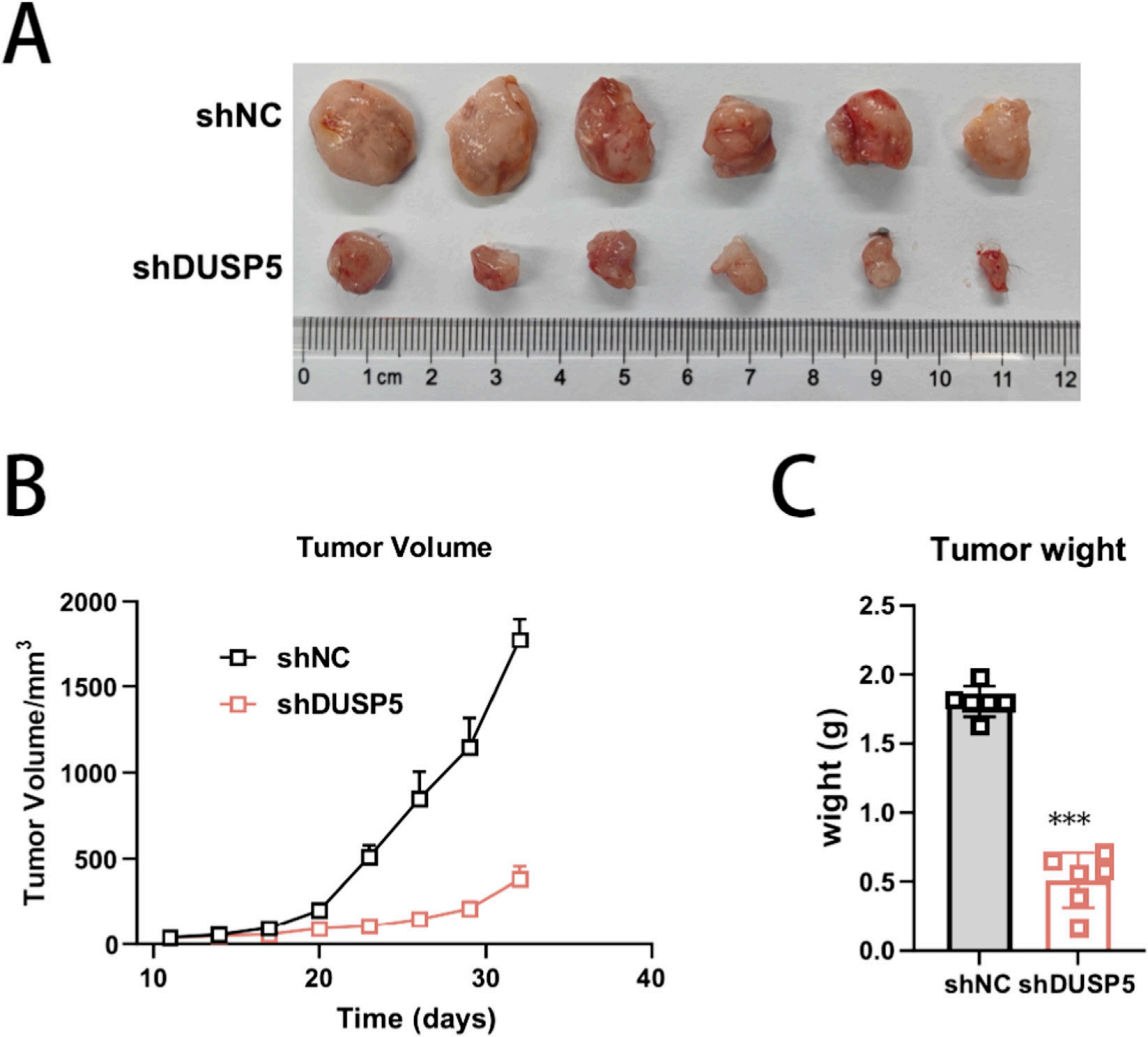
DUSP5 knockdown suppresses tumor growth in an ovarian cancer xenograft model. **(A)** Representative images of subcutaneous tumors derived from shNC or shDUSP5 OVCAR8 cells in nude mice. **(B)** Tumor growth curves showing tumor volume changes over time in the control and DUSP5 knockdown groups. **(C)** Final tumor weights measured at the experimental endpoint. Data are presented as mean ± SD (n = 6). ***P < 0.001 versus shNC.

To further evaluate the role of DUSP5 in ovarian cancer progression *in vivo*, a subcutaneous xenograft model was established using OVCAR8 cells with stable DUSP5 knockdown, with six mice included in each group. Representative images of excised tumors showed that tumors derived from shDUSP5 cells were markedly smaller than those from shNC controls (Figure 12A). Consistently, tumor growth curve analysis demonstrated that DUSP5 knockdown significantly inhibited tumor growth over time, resulting in substantially reduced tumor volumes compared with the control group (Figure 12B). At the experimental endpoint, tumor weights in the DUSP5 knockdown group were also significantly lower than those in the shNC group (Figure 12C). These results support a tumor-promoting role of DUSP5 *in vivo*, although maintenance of knockdown in excised tumor tissues was not directly assessed in the present study.

## 4 Discussion

Platinum-based chemotherapy remains the cornerstone of treatment for ovarian cancer; however, the development of chemoresistance continues to be a major challenge in clinical practice (Binju et al., 2019; van Zyl et al., 2018). In this study, we integrated single-cell transcriptomic analysis with bulk multi-omics datasets to explore transcriptional adaptations associated with chemotherapy exposure in ovarian cancer. Our analyses revealed marked heterogeneity among epithelial tumor cells and identified transcriptional states associated with treatment-related stress responses. Through integrative computational analyses, DUSP5 emerged as a candidate regulator associated with these chemotherapy-related epithelial programs (Yin et al., 2025). Functional experiments further showed that DUSP5 knockdown significantly suppressed ovarian cancer cell proliferation, migration, and clonogenic growth, increased sensitivity to carboplatin, and inhibited tumor growth *in vivo*. These findings suggest that DUSP5 may contribute to tumor cell adaptation under chemotherapy pressure.

DUSP5 is a member of the dual-specificity phosphatase family and is known to negatively regulate ERK signaling within the MAPK pathway (Kidger et al., 2017; Rushworth et al., 2014). Previous studies have suggested that DUSP family members participate in multiple aspects of tumor biology, including proliferation, differentiation, and cellular stress responses (Zandi et al., 2022; Kidger and Keyse, 2016). The biological role of DUSP5 appears to be context-dependent and may vary with molecular background, signaling state, treatment pressure, and experimental system. Although a previous study reported that DUSP5 could suppress ovarian cancer progression through IL-33 signaling (Wang et al., 2019), our analyses and functional assays support a tumor-promoting and platinum-resistance-associated role in the models examined here. This difference may reflect variation in molecular subtype, signaling context, treatment pressure, or experimental system. Our findings indicate that DUSP5 is aberrantly expressed in ovarian tumors and is associated with transcriptional programs linked to tumor progression. Functional assays in ovarian cancer cell lines further support a role for DUSP5 in maintaining tumor cell growth and migratory capacity.

Another important observation from this study is the association between DUSP5 expression and pathways related to epithelial–mesenchymal transition, inflammatory signaling, hypoxia, and metabolism-related tumor programs (Leung et al., 2022; Li et al., 2019). These biological processes have been widely implicated in tumor progression and treatment resistance. EMT, for example, has been linked to increased tumor cell plasticity and the acquisition of drug-resistant phenotypes. Hypoxia, inflammatory signaling, and metabolic remodeling can also influence tumor cell survival and reshape the tumor microenvironment during therapy (Zhang et al., 2025; Zhao et al., 2023b; Wang et al., 2021). Temporal regulation and amino acid metabolism have likewise been connected with immune-state remodeling and therapy-relevant tumor behavior, reinforcing the possibility that DUSP5-associated signatures reflect an adaptive transcriptional state rather than a single isolated pathway event (Yan et al., 2024; Wang et al., 2024). In our analyses, tumors with higher DUSP5 expression showed enrichment of several pathways related to these processes, suggesting that DUSP5 may be involved in broader transcriptional programs associated with tumor adaptation.

In addition to tumor-intrinsic pathways, our analyses also indicated potential associations between DUSP5 expression and immune and stromal features of the tumor microenvironment. This relationship is biologically plausible because chemotherapy can alter the immune landscape of high-grade serous ovarian cancer, and ovarian cancer studies have linked extracellular-vesicle signaling and immune-low spatial states to therapeutic context (Gong et al., 2023; Yan et al., 2025; Vanguri et al., 2022). More broadly, computational immune-gene models and studies of immunomodulatory treatment strategies support interpreting candidate biomarkers together with immune infiltration and treatment context (Li et al., 2023; Huang et al., 2023; Chen et al., 2025). Although the present study primarily focused on tumor cell transcriptional programs, the observed associations raise the possibility that DUSP5 may be involved in treatment-associated microenvironment remodeling. Further studies will be required to clarify the precise mechanisms through which DUSP5 may influence tumor–microenvironment interactions.

Several limitations should also be considered. First, although integrative analyses of public datasets provided valuable insights, additional clinical cohorts with standardized treatment and outcome annotations will be necessary to further validate the clinical relevance of DUSP5. Second, CellChat and immune-infiltration analyses provide computationally inferred or association-based evidence and do not directly demonstrate functional ligand–receptor signaling or immune/stromal remodeling. Third, while our functional experiments confirmed the role of DUSP5 in tumor cell proliferation, migration, and drug sensitivity, most functional validation was performed in ovarian cancer cell lines and a subcutaneous xenograft model rather than immune- or stromal-inclusive systems. Fourth, DUSP5 knockdown was validated before xenograft implantation, but its maintenance in excised tumor tissues was not directly assessed. Future studies incorporating rescue experiments, pathway-intervention assays, immune-competent models, organoid-based platforms, and larger clinical cohorts will help clarify the mechanistic and translational role of DUSP5 in chemotherapy resistance (Zhang et al., 2025).

## 5 Conclusion

In summary, this study provides an integrative analysis of chemotherapy-associated tumor adaptation in ovarian cancer by combining single-cell transcriptomics with bulk multi-omics datasets. Our results identify DUSP5 as a candidate regulator associated with transcriptional programs linked to tumor progression and platinum resistance. Functional experiments further demonstrate that suppression of DUSP5 inhibits tumor cell growth and enhances sensitivity to carboplatin. These findings suggest that DUSP5 may represent a candidate biomarker and therapeutic target for improving platinum responsiveness in ovarian cancer, pending further mechanistic and clinical validation.

## Data Availability

The original contributions presented in the study are included in the article/supplementary material, further inquiries can be directed to the corresponding author.
